# Pulmonary cryptococcosis complicated with pulmonary aspergillosis: a series of studies and a literature review

**DOI:** 10.1186/s12879-024-09014-8

**Published:** 2024-01-16

**Authors:** Xidong Wang, Shaoqiang Li, Mangui Zhu, Ye Qiu, Yilei Hui, Yongming Li, Yangqing Zhan, Yan Wang, Feng Ye, Zhengtu Li

**Affiliations:** 1 State Key Laboratory of Respiratory Disease, National Clinical Research Center for Respiratory Disease, Guangzhou Institute of Respiratory Health, the First Affiliated Hospital of Guangzhou Medical University, 151 Yanjiang Xi Road, Guangzhou, Guangdong 510120 China; 2 Shunde Hospital Affiliated to Guangzhou University of Chinese Medicine, FoShan, 528000 China

**Keywords:** Pulmonary cryptococcosis, Pulmonary aspergillosis, Complicated infection, Voriconazole, Metagenomics next generation sequencing

## Abstract

**Background/Objective:**

With the development of society, pulmonary fungal diseases, represented by pulmonary aspergillosis and pulmonary cryptococcosis, have become increasingly common. However, there is a lack of clear understanding regarding coinfection by these two types of fungi in immunocompetent individuals.

**Methods:**

A retrospective study from 2014 to 2022 and a systematic literature review of original articles published in English were performed. Patients with pulmonary cryptococcosis complicated with pulmonary aspergillosis including 5 in the retrospective study and 6 in the systematic literature review.

**Result:**

The diagnosis of concurrent pulmonary cryptococcosis and pulmonary aspergillosis in patients was confirmed through repeated biopsies or surgical resection. Pulmonary cryptococcosis is often diagnosed initially (6/11, 55%), while the diagnosis of pulmonary aspergillosis is established when the lesions become fixed or enlarged during treatment. Transbronchial lung biopsy (3/11, 27%), thoracoscopic lung biopsy (2/11, 18%), and percutaneous aspiration biopsy of the lung (1/11, 9%) were the main methods to confirm concurrent infection. Most patients were treated with voriconazole, resulting in a cure for the coinfection (6/11, 55%).

**Conclusion:**

Pulmonary cryptococcosis complicated with pulmonary Aspergillus is an easily neglected mixed fungal infection. During the treatment of lesion enlargement in clinical cryptococcus, we need to watch out for Aspergillus infection.

## Introduction

With the development of modern medical and health care, the large-scale use or abuse of broad-spectrum antibiotics, glucocorticoids, and immunosuppressants, the rise of organ transplantation technology, and the increasingly diverse means of tumour radiotherapy and chemotherapy have brought benefits to human beings but have also brought new crises [1]. According to a survey of the prevalence of fungal diseases in multiple countries, more than 3 million people a year now contract pulmonary mycosis, a 41-fold increase in 32 years [2]. In 2022, the World Health Organization (WHO) released the list of health-threatening fungi for the first time, and many opportunistic pathogenic fungi entered human view. *Aspergillus fumigatus*, *Candida auris*, *Cryptococcus neoformans* and *Candida albicans* were listed as the Critical Priority Group in the fungal list, among which Aspergillus fumigatus and Candida auris mainly cause respiratory infections [3].

As fungi that are extensively tested in clinical settings, both Aspergillus and Cryptococcus can cause primary infections of a single organ (usually the lung) or disseminated infections resulting in multiorgan dysfunction, such as cryptococcal meningitis and invasive pulmonary aspergillosis [4]. Even in immunocompetent patients, these infections have a high mortality rate [5]. Therefore, accurate diagnosis and timely antifungal therapy are particularly important to avoid a misdiagnosis or a missed diagnosis, which may result in death or progression to severe chronic disease.

Reports of single opportunistic pathogenic fungal infections causing pneumonia are common, while studies on coinfection of fungi are lacking. Although the incidence of coinfection of fungal disease is not high, its clinical presentation is nonspecific and lacks sufficiently sensitive noninvasive diagnostic methods, which may lead to underestimation of its incidence and make it prone to a misdiagnosis and a missed diagnosis. Reports of coinfection of Aspergillus and Cryptococcus are rare, and their characteristics have not been described in detail. This study aims to provide clinical experience for the early diagnosis and standardized treatment of this disease by reporting a series of cases while reviewing previous reports on coinfection of Aspergillus and Cryptococcus.

## Method

### Retrospective study

A retrospective study was conducted on patients diagnosed with pulmonary cryptococcosis and pulmonary aspergillosis who were HIV-negative. The time range was from January 1st, 2014, to December 31st, 2022. All patients provided informed consent and received support from the First Affiliated Hospital of Guangzhou Medical University.

### Systematic review

#### Search strategy

**Fig. 1:**
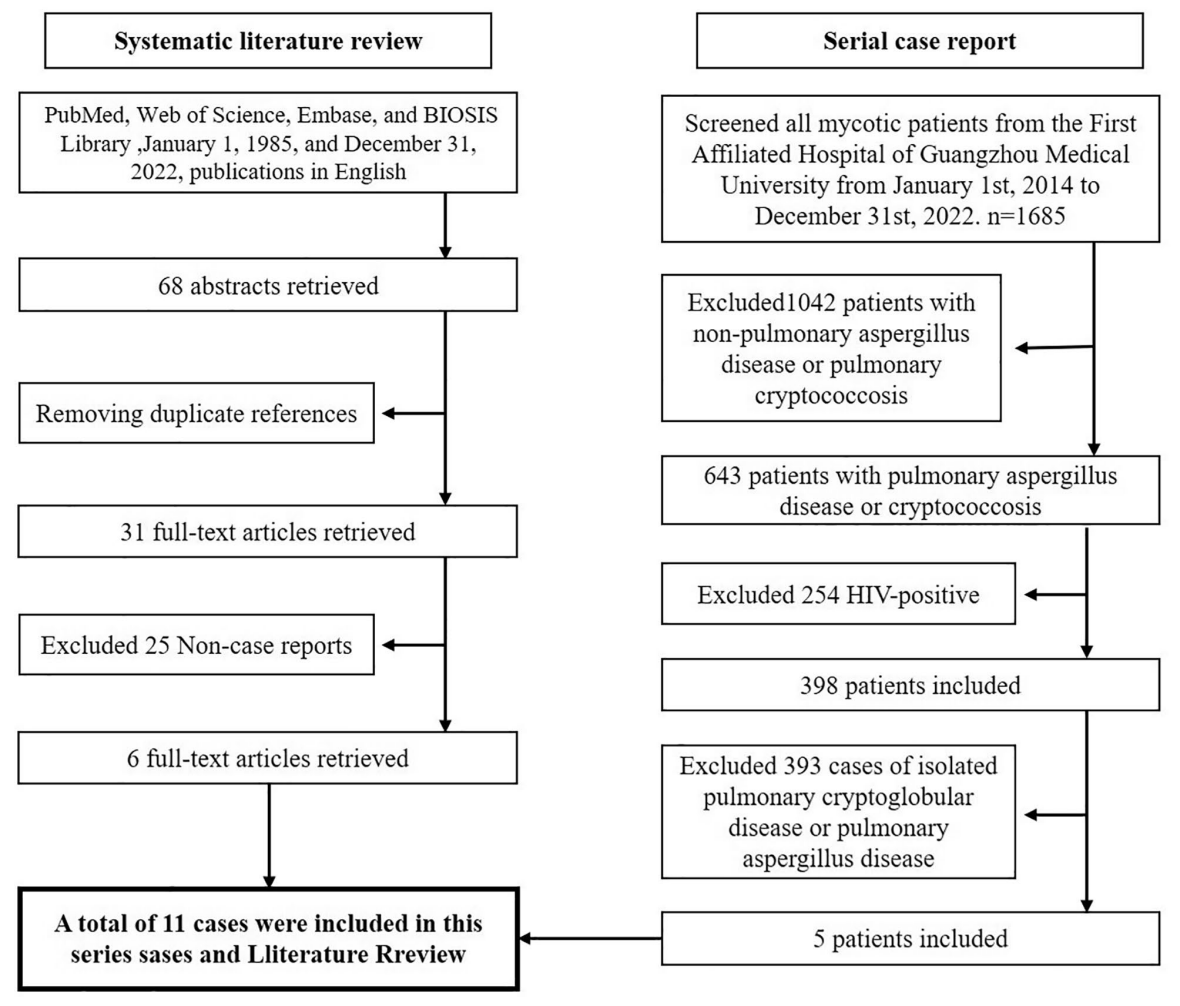
Flow chart of the literature included in the review and the selected cases

We searched PubMed, Web of Science, Embase, and BIOSIS Library for 68 original case reports and cohort studies on pulmonary cryptococcosis with pulmonary aspergillosis published in English between January 1, 1985, and December 31, 2022. We used the keywords “Aspergillus and cryptococcus and coinfection,” “Coinfection with cryptococcus and aspergillus,” “((cryptococcus) AND (aspergillus)) AND (coinfection),” and “Pulmonary cryptococcosis with pulmonary aspergillosis.” The references of the retrieved articles were reviewed for additional relevant citations. The abstracts of all identified articles were viewed, and the full-text versions of relevant articles were retrieved for data extraction and analysis [6–11]. (Fig. 1)

### Pulmonary cryptococcosis complicated with pulmonary aspergillosis definition

Histopathological examination of pathogenic bacterial infection is the diagnostic criterion [12]. Positive results can be obtained from histopathological, cytological, or direct microscopic examination of normally sterile abnormal sites that are clinically or radiographically consistent with an infectious disease process, using aseptic techniques to obtain specimens (excluding bronchoalveolar lavage fluid, BALF), or from culture-positive/fungal DNA amplified by PCR and DNA sequencing in formalin-fixed, paraffin-embedded tissues where fungi are found. Invasive pulmonary fungal disease requires at least one host factor, one clinical feature, and one mycological evidence and is only applicable to immunodeficient patients. The diagnosis comes from the 2019 EORCT/MSGERC Guidelines for Invasive Mycosis. Mycological evidence is an important basis for clinical diagnostic criteria for pulmonary aspergillosis and pulmonary cryptococcosis, which can be in the sputum, BALF, etc., or from the culture of any mould and the microscopic detection of any suggestive mould components. For Aspergillus disease, GM test plasma, serum, BAL detected antigen or titre ≥ 1.0, plasma titre ≥ 0.7, or BALF titre ≥ 0.8 can be used. Continuous positive PCR test results can also lead to a clinical diagnosis.

### Inclusion and exclusion criteria

Inclusion Criteria: (1) HIV-negative patients with pulmonary cryptococcosis complicated with pulmonary aspergillosis as defined above were included for analysis. (2) Original studies reporting patients with pulmonary cryptococcosis complicated with pulmonary aspergillosis as defined above were included for analysis. (3) Informed consent was obtained from the patients.

The exclusion criteria were as follows: (1) articles on mycology, diagnostics, antifungal susceptibility and experimental animal studies; (2) general review articles on pulmonary cryptococcosis and pulmonary aspergillosis; and (3) case reports or series involving HIV-positive individuals. For duplicate publications, the most recent article was used for data extraction.

## Result

### Demographic and clinical characteristics of patients with pulmonary cryptococcosis complicated with pulmonary aspergillosis

In this retrospective study and literature review, we investigated 11 patients with pulmonary cryptococcosis complicated with pulmonary aspergillosis. The study focused on HIV-negative patients with pulmonary cryptococcosis complicated with pulmonary aspergillosis. The patients in this retrospective study were from the First Affiliated Hospital of Guangzhou Medical University, and a total of 5 patients were included. Four patients underwent histological examination of bronchoscopy biopsy or percutaneous lung puncture, and the remaining patients underwent histological examination after pulmonary lobectomy. There were 6 cases from 6 case reports after excluding systematic reviews and duplicate reports. Among the 11 patients, 7 were from China, 1 was from Taiwan, 2 were from the United States, and 2 were from Japan. There were 3 female and 8 male patients, with 7 patients in the younger age group (18–44 years), 2 patients in the middle-aged group (45–59 years), and 2 patients in the elderly group (60–74 years), with a median age of 38 (31–74 years).

Cough (6/11), fever (3/11), sputum (2/11), haemoptysis (2/11), and chest pain (2/11) were the most common clinical symptoms. One patient had gastrointestinal symptoms of anorexia, and another patient had weight loss. Notably, 3 patients did not have any clinical symptoms and were found to have pulmonary shadows or cavities during physical examination, prompting them to seek medical attention. Four patients were diagnosed with both pulmonary cryptococcosis and pulmonary aspergillosis through histological evidence. Six patients were initially diagnosed with pulmonary cryptococcosis and later diagnosed with pulmonary aspergillosis after treatment, and 1 patient was diagnosed clinically. The remaining 10 patients were diagnosed with invasive methods, including transbronchial lung biopsy, CT-guided lung puncture biopsy, VATS lung biopsy, and surgical lung resection, with pulmonary tissue pathology confirming the diagnosis [6–11] (Table 1).

**Table 1:** Basic patient information

NO.	Country	Age	Gender	cardinal symptom	Underlying diseases/Drug use	Therapeutic process	Ultimate treatment	Prognosis	Means of diagnosis
**P1**	China	50	Female	Cough, Bloodshot sputum	Diabetes	Pulmonary cryptococcosis → Fluconazole 6 month (poor response) → right lower lobe resection. Pathology: Cryptococcus + Aspergillus	Voriconazole	Cure	Thoracoscopic lobectomy
**P2**	China	38	Male	Physical examination revealed lung shadow	/	Pulmonary cryptococcosis → Voriconazole 5 m (no absorption) to Fluconazole 3 m (no absorption) → Pathology slide consultation: Cryptococcus + Aspergillus	Voriconazole	Cure	TBLB
**P3**	China	35	Male	Cough, Phlegm, Fever	Horseshoe kidney	Pulmonary cryptococcosis → Fluconazole 10 m (first reduced and then increased) →pathology: Aspergillus	Voriconazole	Cure	TBLB + CT-guided percutaneous lung biopsy
**P4**	China	53	Male	Physical examination revealed a cavity in the right lung	Diabetes	Pulmonary cryptococcosis → Fluconazole 2 m (first reduced lesion, then no change) → Pathology: pulmonary Aspergillus with cryptococcus	Voriconazole	Cure	TBLB
**P5**	China	32	Male	Physical examination revealed a shadow of the lower left lung	/	Pulmonary cryptococcosis → Fluconazole (lesion enlargement)→ Pathology: pulmonary Aspergillus with cryptococcus	Voriconazole	Improvement	TBLB
**P6**	America^16^	31	Male	Chest pain, Dry cough, Night sweats	/	Pulmonary cryptococcosis + Aspergillus disease → amphotericin B (cavity enlargement) → lobectomy → amphotericin B	Amphotericin B + lobectomy	Improvement	Lobectomy
**P7**	China^15^	33	Female	Chest pain, Fever	Systemic lupus erythematosus (glucocorticoid hormone + azathioprine)	Pulmonary aspergillosis + pulmonary cryptococcosis → amphotericin B + Fluconazole	Amphotericin B + Fluconazole	Improvement	VATS lung biopsy
**P8**	Japan^14^	74	Male	Dry cough	Tuberculosis	Suspected malignancy of the left inferior void → lobectomy → pathology: pulmonary aspergillosis + pulmonary cryptococcosis	Not mentioned	Not mentioned	Thoracoscopic lobectomy
**P9**	Japan^13^	64	Male	Two cavities in the lungs with aspergillus pellets	Rheumatoid arthritis (glucocorticoid + cyclophosphamide)	TBLB was used to diagnose Cryptococcus pneumoniae with fungal spherules → Fluconazole →VATS, and PCR confirmed cryptococcus with aspergillus infection	Fluconazole	Not mentioned	VATS lung biopsy
**P10**	China^12^	33	Male	Haemoptysis	/	Empiric anti-infective + anti-tuberculosis therapy 1 week (no improvement in symptoms) →VATS left lower lobectomy → Pathology: pulmonary Aspergillus + Cryptococcus pulmonary	Amphotericin B + Fluconazole	Cure	Thoracoscopic lobectomy
**P11**	America^11^	69	Female	Dyspnoea, Fever, Dry cough, Loss of appetite, Fatigue	Post-bone marrow transplantation	Pulmonary cryptococcosis → Flucytosine + amphotericin B liposomes (aggravation of symptoms and CT findings)	Voriconazole	Improvement	Cryptococcal antigen titre 1:640; BALF-GM test was 3.258

### Laboratory and radiographic characteristics of patients with pulmonary cryptococcosis complicated with pulmonary aspergillosis

**Fig. 2:**
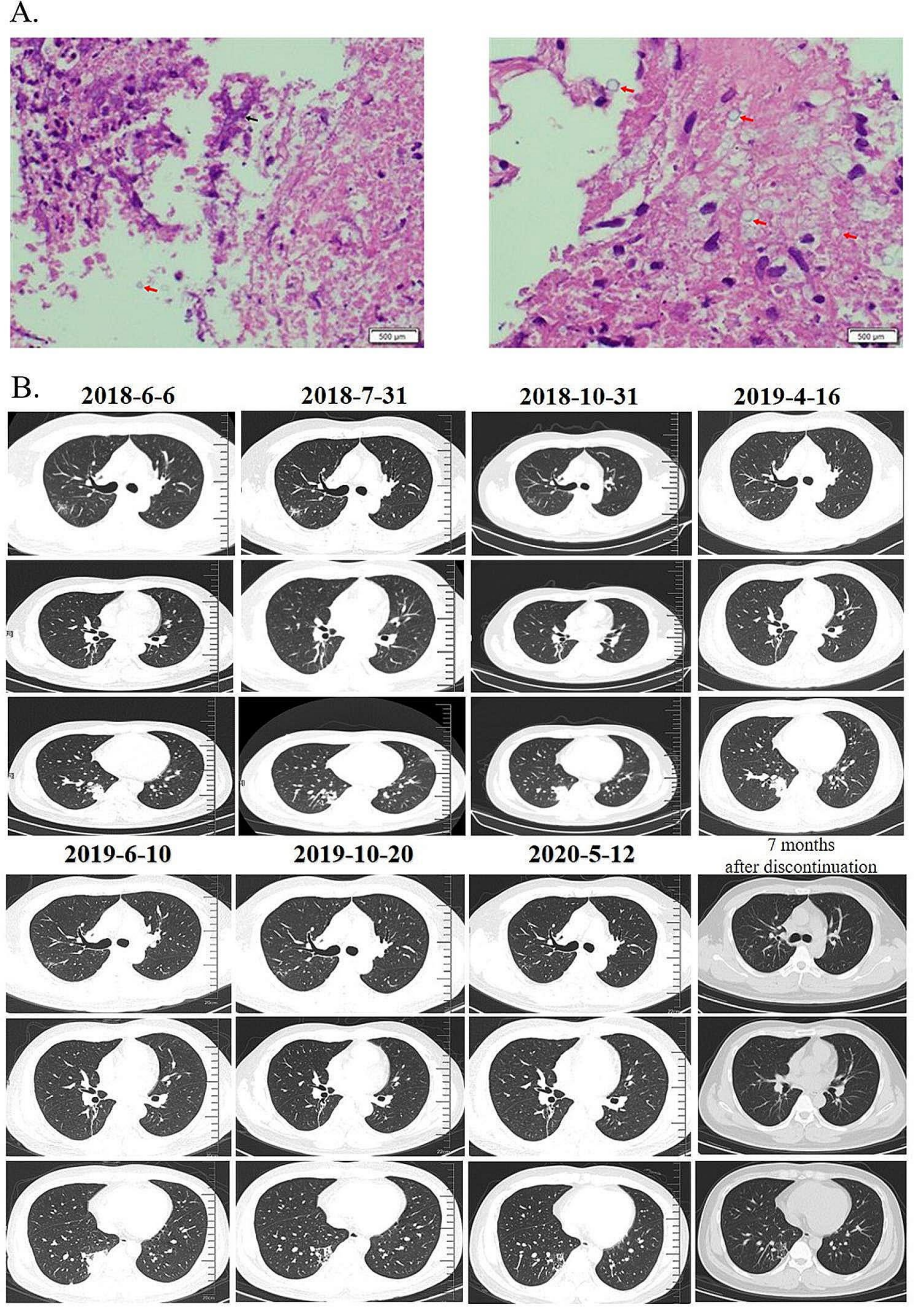
Histopathological findings and CT results of the patient. **A**: From P2, found in a single tissue sample with cryptococcus and Aspergillus infection. The lung tissue sent for examination showed foci of coagulative necrosis, in which refractory strong spores were seen, and neutrophilic exudates, abscess formation, in which sharply branched hyphae and spores were also seen, and eosinophils and lymphocyte infiltration in the adjacent lung tissue. The histologic changes were those of pulmonary cryptococcosis combined with Aspergillus infection. **B**: The patient’s CT results are arranged in chronological order. The patient’s CT results are arranged in chronological order. The sequence is as follows: initial diagnosis of pulmonary cryptococcosis, diagnosis of pulmonary cryptococcosis combined with pulmonary aspergillosis infection, and postcure imaging

Routine blood examination showed that 6/7 patients had a normal white blood cell count, 5/5 had a normal neutrophil count, and 5/5 had a normal lymphocyte count. A total of 5/8 patients had positive serum cryptococcal antigen titres, while 2/8 had positive Aspergillus antigen titres. Seven patients tested negative for the G test, 3 patients did not have G test results mentioned, and 1 patient was not tested for fungal infection at the time and did not have a G test performed. 50% (2/4) of the patients had positive cryptococcal antigen titres in bronchoalveolar lavage fluid, while 2/5 had positive Aspergillus antigen titres. Microbial cultures were positive in 3/11 of the patients, with specimens including sputum, bronchoalveolar lavage fluid, and sputum smears. Partial pathogenic bacteria were detected in 2 patients (2/11) using metagenomic next-generation sequencing (mNGS) technology, with Aspergillus detected in one patient and cryptococcus detected in another (Table 2; Fig. 2A).

**Table 2:** Laboratory examination and microbiological testing

No.	WBC	NEU	LYM	Cryptococcus antigen test (serum)	Cryptococcus antibody		Aspergillus antigen		aspergillus antibody	G test	Cryptococcus antigen test (BALF)	GM test (BALF)	Pathogen culture	mNGS	Histopathological examination	Initial diagnosis and basis	Final diagnosis and basis
**P1**	7	3.8	2.5	negative	/	negative		/		negative	undetected	undetected	Sputum + BALF culture: Cryptococcus neoformans	/	Lung tissue changed to cryptococcus + Aspergillus infection	**Pulmonary cryptococcosis** Sputum + BALF culture: Cryptococcus neoformans	**Pulmonary cryptococcosis complicated with pulmonary aspergillosis** Lung tissue changed to cryptococcus + Aspergillus infection
**P2**	7	3.0	3.2	negative	/	negative		/		negative	< 3.2(negative)	0.267(negative)	negative	/	Lung tissue showed pulmonary cryptococcosis with aspergillus infection	**Pulmonary cryptococcosis** Lung tissue showed pulmonary cryptococcosis	**Pulmonary cryptococcosis complicated with pulmonary aspergillosis** Lung tissue showed pulmonary cryptococcosis with aspergillus infection
**P3**	6.98	3.6	2.6	> 100.0 ug/L	/	negative		/		negative	8.701 µg/L	negative	BALF culture: Cryptococcus neoformans	Lung Tissue: Aspergillus fumigatus	Lung tissue showed chronic bronchitis with aspergillus infection	**Pulmonary cryptococcosis** BALF culture: Cryptococcus neoformans and Cryptococcus antigen test (BALF): 8.701 µg/L	**Pulmonary aspergillosis** Lung Tissue: Aspergillus fumigatus and Lung tissue mNGS showed aspergillus infection
**P4**	9.9	5.8	2.8	negative	/	negative		/		negative	9.329 µg/L	0.309 µg/L(negative)	negative	/	Lung tissue showed lesions consistent with pulmonary aspergillus with cryptococcus infection	**Pulmonary cryptococcosis** Cryptococcus antigen test (BALF): 9.329 µg/L	**Pulmonary cryptococcosis complicated with pulmonary aspergillosis** Lung tissue showed lesions consistent with pulmonary aspergillus with cryptococcus infection
**P5**	5.7	3.4	1.6	positive	/	2.44 µg/L		/		negative	negative	2.0479	negative	BALF: Cryptococcus neoformans	Lung tissue showed pulmonary Aspergillus with cryptococcus infection	**Pulmonary cryptococcosis** Cryptococcus antigen test(serum): positive, BALF culture: Cryptococcus neoformans	**Pulmonary cryptococcosis complicated with pulmonary aspergillosis** Lung tissue showed pulmonary cryptococcosis with aspergillus infection
**P6**	unreported	unreported	unreported	1:2	positive	unreported		/		unreported	unreported	unreported	Sputum smear: Cryptococcus; Sputum culture: Aspergillus fumigatus + Candida albicans	/	Lung tissue changed to cryptococcus + Aspergillus infection	**Pulmonary cryptococcosis complicated with pulmonary** Lung tissue changed to cryptococcus + Aspergillus infection	**Co-infection diagnosis was made at once**
**P7**	unreported	unreported	unreported	unreported	/	unreported		/		unreported	unreported	unreported	Lung tissue culture: Aspergillus flavus	/	Wrapped Cryptococcus yeast cells and entwined aspergillus filaments were seen in lung tissue	**Pulmonary cryptococcosis complicated with pulmonary** Wrapped Cryptococcus yeast cells and entwined aspergillus filaments were seen in lung tissue	**Co-infection diagnosis was made at once**
**P8**	unreported	unreported	unreported	undetected	/	undetected		/		undetected	undetected	undetected	Lung tissue culture: Aspergillus and Cryptococcus neoformans	/	The lung tissue showed positive anti-aspergillus antibody for cryptococcus and isolated hypha	**Suspected malignancy of the left inferior void**	**Pulmonary cryptococcosis complicated with pulmonary aspergillosis** The lung tissue showed positive anti-aspergillus antibody for cryptococcus and isolated hypha
**P9**	13.4	unreported	unreported	positive	/	negative		negative		negative	undetected	undetected	Lung tissue culture: Cryptococcus neoformans	/	A ball of fungus + cryptococcus	**Pneumoniae cryptococcosis with fungal spherules** A ball of fungus + cryptococcus	**Pulmonary cryptococcosis complicated with pulmonary aspergillosis** A ball of fungus + cryptococcus And PCR confirmed Aspergillus infection
**P10**	unreported	unreported	unreported	unreported	/	negative		/		negative	unreported	unreported	negative	/	Two fungal lesions in lung tissue, one was cryptococcal lesion, the other was aspergillus lesion	**Undefined**	**Pulmonary cryptococcosis complicated with pulmonary aspergillosis** Two fungal lesions in lung tissue, one was cryptococcal lesion, the other was aspergillus lesion
**P11**	unreported	unreported	unreported	1: 640	/	negative		/		unreported	unreported	3.258	negative	/	**/**	**Pulmonary cryptococcosis** Cryptococcal antigen test 1:640	**Pulmonary cryptococcosis complicated with pulmonary aspergillosis** Cryptococcal antigen test 1:640 BALF-GM test was 3.258 And Flucytosine + amphotericin B liposomes treat aggravation of symptoms and CT findings

CT showed no lesion change in 2 patients after the initial treatment, lesion enlargement in 2 patients after treatment, first reduction and then enlargement in 1 patient, and unchanged lesion in 1 patient after reduction. Five of the 11 patients had multiple nodules or mass shadows, and 1 patient had a mass-like density increase, consistent with typical CT manifestations of pulmonary aspergillosis or pulmonary cryptococcosis. Nine of the 11 patients had radiographic findings that showed cystic lesions, with 2 patients showing thick-walled cavities, one of which had a spike, and 2/11 showed nodular cavity lesions, consistent with the typical CT manifestations of pulmonary aspergillosis. A total of 3/11 lesions were concentrated under the pleura, presenting as infiltrative shadows or nodular cavities. The CT characteristics of 5 patients from our hospital showed that the lesion was initially considered cryptococcus, which was partially absorbed after anti-cryptococcus therapy. After further treatment, the lesion was enlarged and finally diagnosed as complicated with Aspergillus infection (Fig. 2B).

### Treatment and outcome of pulmonary cryptococcosis complicated with pulmonary aspergillosis

There were 11 patients with pulmonary cryptococcosis complicated with pulmonary aspergillosis. Among them, five were initially treated with fluconazole, which was partially effective, but then there was either an increase in lesions or no change. One patient underwent a lobectomy after the initial treatment was ineffective, and histopathology confirmed mixed infection of Cryptococcus and Aspergillus. The patient was cured after receiving voriconazole treatment for one year. One patient was initially treated with voriconazole for 5 months without improvement and then switched to fluconazole for 3 months without improvement. After histopathological diagnosis, the patient was treated with voriconazole for one year and was cured. (Patients with liver enzyme CYP2C9 enzyme activity had the high, fast metabolic type. The blood concentrations of previous medication were not monitored, and voriconazole did not reach the therapeutic dose.)Three patients were initially treated with amphotericin B. One patient was treated with amphotericin B alone after a diagnosis of a mixed infection with Cryptococcus and Aspergillus, but the lesions increased. The patient underwent a lobectomy and continued to receive amphotericin B treatment, resulting in improvement. One patient was treated with a combination of flucytosine and amphotericin B. After one month of treatment with fluconazole instead of flucytosine on the basis of no involvement of the central nervous system, antifungal drugs were discontinued. Two weeks later, the patient’s symptoms and imaging worsened, and voriconazole was used instead, resulting in improvement. One patient was directly treated with a combination of amphotericin B and fluconazole after a diagnosis of a mixed infection consisting of Cryptococcus and Aspergillus. One patient underwent a lobectomy after routine anti-infection and anti-tuberculosis treatment was ineffective and was then treated with amphotericin B and fluconazole after surgery, resulting in cure. One patient was directly treated with lobectomy. Four patients had effective treatment with antifungal drugs after excision of the lesion tissue by lobectomy. Voriconazole monotherapy was mostly effective, with a cure/improvement rate of up to 54.5% (6/11) (Table 1).

## Discussion

In this study, we included 11 cases of combined pulmonary cryptococcosis and aspergillosis, which are uncommon globally. Reports mainly focus on immunocompromised hosts or patients using immunosuppressive agents. We reviewed 5 cases of pulmonary cryptococcosis combined with aspergillosis in our hospital, reflecting the emerging trend of pulmonary fungal diseases in immunocompetent hosts. These patients were all from the immunocompetent population, with an average age of 42 years, ranging from 32 to 53 years old, and were mainly middle-aged. Three of them were screened for the disease during physical examination and showed no clinical symptoms. All 5 patients were initially diagnosed with pulmonary cryptococcosis. After treatment, which was primarily with fluconazole, the lesions showed varying degrees of improvement but did not disappear completely. In some patients, the lesions even increased in size. Subsequent rebiopsy confirmed the diagnosis of pulmonary cryptococcosis complicated by pulmonary aspergillosis. The patient recovered after treatment with voriconazole. Therefore, the appearance of fixed or unusually enlarged lesions during the treatment of pulmonary cryptococcosis should raise awareness of the possible presence of concomitant pulmonary aspergillosis.

Both cryptococcosis and aspergillosis are common fungal infections in the respiratory system. Compared to cryptococcosis, the disease classification of aspergillosis is more complex [13]. However, the clinical symptoms of both infections are similar and are mainly respiratory symptoms such as cough, sputum production, shortness of breath, and chest pain (Table 1). aspergillosis mostly lack specific clinical manifestations. Typical CT manifestations include cavities, as well as clear crescent or halo signs. However, early presentations are often dominated by small nodules or granulomas [14]. Cryptococcosis infection is often more localized, with cough, sputum production, and chest tightness being the main clinical manifestations. The CT manifestation is mostly single or multiple nodules or consolidation shadows, with visible bronchial meteorology in the consolidation [15]. Single-fungus lung infections are common in both immunocompetent and immunodeficient populations, and appropriate diagnosis and treatment options are available. However, there are very few reports of mixed fungal infections in immunocompetent populations. The diagnosis of mixed infections is also a cumbersome process. All 5 patients in our centre had improved their symptoms after the first treatment of pulmonary cryptococcal disease, but patients’ clinical symptoms did not completely disappear (Table 1). Even in patients without clinical symptoms at the first diagnosis, clinical symptoms such as cough, sputum production, and chest pain appear. There is a correspondence between clinical symptoms and CT findings at this time point. However, the presence of coinfection cannot be determined from this, and a more detailed diagnostic process is required to make this judgement.

The diagnosis of PC and IC can be challenging, and a comprehensive evaluation including laboratory and radiologic tests is necessary for accurate diagnosis and prompt treatment. Unlike previous reports where the pathological conclusion of mixed infection of pulmonary cryptococcosis and aspergillosis was obtained through lung lobectomy or thoracoscopic lung biopsy after initial empirical treatment was ineffective, in this study, 3 out of 5 patients had pulmonary tissue collected at the lesion site through transbronchial lung biopsy (TBLB). The pulmonary tissue was clearly diagnosed as combined pulmonary cryptococcosis and aspergillosis (Table 1; Fig. 2). One patient was diagnosed through percutaneous lung puncture biopsy guided by CT, and only one patient had to undergo lung lobectomy after the initial fluconazole treatment was ineffective due to the lesion being limited to the lower right lung and fungal culture from bronchoalveolar lavage fluid (BALF) revealing the *C. neoforman*s strain. According to research, compared with thoracoscopic lung biopsy techniques, endoscopic operations through bronchoscopy cause less damage to patients, resulting in a lower probability of pathogenic bacterial dissemination and a relatively better differential effect for the three types of diseases: lung cancer, pulmonary tuberculosis, and pulmonary fungal disease [16]. Meanwhile, percutaneous lung puncture guided by CT can compensate for the blind spots of bronchoscopic lung biopsy in detecting the lower branches of the pulmonary bronchi or peripheral areas [17].

Triazole antifungal drugs such as fluconazole and voriconazole and polyene antifungal drugs such as amphotericin B play an important role in the treatment of pulmonary fungal diseases. In this study, patients presented with nodules, cavities, and increased density in the lungs on CT scans, as well as laboratory tests or histological evidence of cryptococcal infection. The majority of patients were initially diagnosed with pulmonary cryptococcosis, and fluconazole was chosen as the first-line treatment for local therapy, while amphotericin B plus flucytosine was selected as the induction therapy. This treatment covers most Candida and Cryptococcus species but lacks coverage for concomitant Aspergillus infection. As the most common pulmonary fungal infection, pulmonary aspergillosis is associated with high in-hospital mortality rates and prolonged hospital stays, with up to a 40% higher risk of readmission and a high mortality rate (30–60%) for invasive infections [18, 19]. After diagnosing concomitant aspergillosis, patients were given the option of using voriconazole, which resulted in improvement or cure. Voriconazole has a broader spectrum of antibacterial activity than fluconazole, covering Cryptococcus, Aspergillus, and Candida species [20]. Although it is not a first-line drug for neonatal cryptococcosis, it is an effective alternative to fluconazole for improving survival in immunosuppressed animal experiments [21]. Voriconazole is also a potential treatment option for induction therapy in cryptococcal central nervous system infections [22]. In patients with refractory pulmonary cryptococcosis, switching from fluconazole to voriconazole can achieve good therapeutic effects [23]. surgery was not necessary, However, among the 11 patients, some underwent surgery followed by a period of antifungal medication.

If early pathological diagnosis confirms a mixed infection of pulmonary cryptococcosis and pulmonary aspergillosis, there is no difficulty in clinical diagnosis. However, in some cases, patients may initially be diagnosed with pulmonary cryptococcosis, and after a period of treatment, they may experience improvement followed by deterioration or no significant improvement at all. In such situations, further differential diagnosis is needed. Delays in the diagnosis of pulmonary cryptococcosis combined with aspergillosis can cause serious adverse consequences. Cryptococcus breaking through the blood‒brain barrier and the invasive infectious nature of pulmonary aspergillosis often lead to poor prognosis [24]. Therefore, a certain diagnostic process needs to be followed to ensure that patients receive a timely diagnosis of pulmonary cryptococcosis combined with aspergillosis. The following are the differential diagnosis directions we need to consider. Is there drug resistance? Is there immune reconstitution inflammatory syndrome, Tumour, or a Mixed infection? When patients undergo CT and other imaging studies and show symptoms such as patchy density increase, nodular shadows, or cavities in the lungs, evidence of cryptococcal infection can be obtained through bronchoscopy biopsy, percutaneous lung puncture, deep sputum culture, or direct microscopic examination. Certainly, considering the complexity of the patients’ conditions, we recommend more proactive diagnostic methods, with invasive examinations being preferred.

**Fig. 3:**
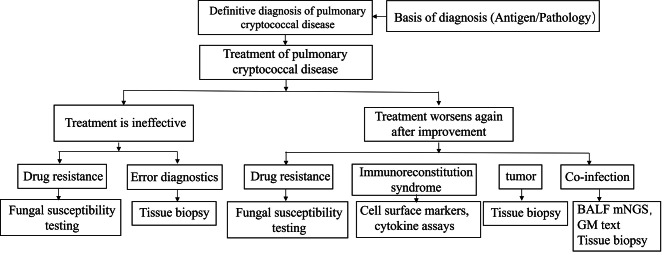
Diagnostic idea diagram of pulmonary cryptococcal disease after initial treatment is ineffective

Then the recommended treatment plan for pulmonary cryptococcosis can be followed according to the guidelines [12, 25]. For example, a classic regimen such as monotherapy with fluconazole or combination therapy of amphotericin B and flucytosine induction can be used. If the lesion does not change or increases again after initial treatment, it is necessary to pay attention to the possibility of drug resistance. In the past decade of widespread use of antifungal drugs, more than 30% of cryptococcus isolates are no longer sensitive to fluconazole [26]. This situation is particularly common in HIV-positive patients, so it is necessary to reconsider the treatment plan based on culture and drug sensitivity results [27]. In addition, the immune reconstitution inflammatory syndrome (IRIS) of HIV-positive patients should not be ignored. In hospitalized HIV-positive patients, the incidence of pulmonary cryptococcosis is 11%. In HIV-infected patients with concomitant pneumonia, the proportion of serum cryptococcal antigen-positive individuals is as high as 13% [28]. Tumours and mixed infections still need to be considered because the presentation of lung cancer and pulmonary fungal disease is similar, but their treatment methods differ significantly, requiring an accurate diagnosis. The imaging features of lung cancer and pulmonary fungal disease are mainly characterized by pulmonary shadows or masses, and laboratory tests often lack specific indicators. Therefore, if there is an increase in laboratory indicators related to lung cancer or if the patient experiences symptoms such as pleural effusion or lung collapse, a more aggressive examination of the pulmonary lesion needs to be carried out again, such as lung biopsy under thoracoscopy, to further clarify the diagnosis (Fig. 3).

In summary, the coexistence of pulmonary cryptococcosis and pulmonary Aspergillus disease is rare but potentially life-threatening in both immunocompetent and immunodeficient patients. Our article also has some shortcomings. The number of patients included in this retrospective study was small, and some clinical data were incomplete. For patients diagnosed first with Cryptococcus and then Aspergillus, it is still unclear whether it is a coinfection or a secondary infection; However, for the first time, this study systematically reviewed the infection characteristics and treatment methods of pulmonary cryptococcosis complicated with concomitant pulmonary aspergillosis at all ages. The aim of this study was to provide a basis for the early diagnosis and accurate treatment of such patients. Further research is needed for optimal management of this rare but important clinical entity.

## Data Availability

At the request of the Ethics Committee, The datasets generated and/or analysed during the current study are available in the Specialist Big Data Management Platform of China, https://copd.tp-data.com/v3/index.html” The Accompanying Key can be obtained by contacting tu276025@gird.cn. Potential conflicts of interest. The authors declare no conflicts of interest.

## References

[1] Wheeler ML, Limon JJ, Underhill DM (2017). Immunity to commensal fungi: Detente and disease [J]. Annu Rev Pathol.

[2] Bongomin F, Gago S, Oladele RO et al. Global and multi-national prevalence of fungal diseases-estimate precision [J]. J Fungi (Basel), 2017, 3(4). 10.3390/jof3040057 PMC5753159 29371573

[3] Who releases first (2022). Ever list of health-threatening fungi [J]. Saudi Med J.

[4] Loughlin L, Hellyer TP, White PL (2020). Pulmonary aspergillosis in patients with suspected ventilator-associated pneumonia in Uk icus [J]. Am J Respir Crit Care Med.

[5] Nguyen MH, Husain S, Clancy CJ (2010). Outcomes of central nervous system cryptococcosis vary with host immune function: results from a multi-center, prospective study [J]. J Infect.

[6] Awari DW, Shah AS, Sexton AM et al. Coinfection of aspergillus and cryptococcus in immunocompromised host: A case report and review of literature [J]. Case Rep Infect Dis, 2020, 2020: 8888270. 10.1155/2020/8888270 PMC7396101 32774953

[7] Wang Q, Wang Z, Hao Y (2018). Coinfection with cryptococcus and aspergillus in an immunocompetent adult: a case report [J]. Med (Baltim).

[8] Kimura M, Maenishi O, Enoki E (2015). Aspergillus fungal ball in central cavity of a pulmonary cryptococcal nodal lesion [J]. Pathol Int.

[9] Enoki E, Maenishi O, Chikugo T (2012). Coinfection of aspergillus and cryptococcus in post-tuberculosis pulmonary cavity [J]. Pathol Int.

[10] Lin CM, Tsai YH, Huang CC (2006). Invasive pulmonary aspergillosis and pulmonary cryptococcosis really coexist in immunocompromised host [J]. J Infect.

[11] Rosenheim SH, Schwarz J (1975). Cavitary pulmonary cryptococcosis complicated by aspergilloma [J]. Am Rev Respir Dis.

[12] Donnelly JP, Chen SC, Kauffman CA (2020). Revision and update of the consensus definitions of invasive fungal disease from the European organization for research and treatment of cancer and the mycoses study group education and research consortium [J]. Clin Infect Dis.

[13] Warris A (2014). The biology of pulmonary aspergillus infections [J]. J Infect.

[14] Raju S, Ghosh S, Mehta AC (2017). Chest ct signs in pulmonary disease: a pictorial review [J]. Chest.

[15] Sui X, Huang Y, Song W (2020). Clinical features of pulmonary cryptococcosis in thin-section ct in immunocompetent and non-aids immunocompromised patients [J]. Radiol Med.

[16] Mondoni M, Rinaldo RF, Carlucci P (2022). Bronchoscopic sampling techniques in the era of technological bronchoscopy [J]. Pulmonology.

[17] Lee SM, Park CM, Lee KH (2014). C-arm cone-beam ct-guided percutaneous transthoracic needle biopsy of lung nodules: clinical experience in 1108 patients [J]. Radiology.

[18] Zilberberg MD, Harrington R, Spalding JR (2019). Burden of hospitalizations over time with invasive aspergillosis in the United States, 2004–2013 [J]. BMC Public Health.

[19] Douglas AP, Smibert OC, Bajel A (2021). Consensus guidelines for the diagnosis and management of invasive aspergillosis, 2021 [J]. Intern Med J.

[20] Malani AN, Kerr LE, Kauffman CA, Voriconazole (2015). How to use this antifungal agent and what to expect [J]. Semin Respir Crit Care Med.

[21] Silva EG, Paula CR, de Assis Baroni F (2012). Voriconazole, combined with amphotericin b, in the treatment for pulmonary cryptococcosis caused by c. Neoformans (serotype a) in mice with severe combined immunodeficiency (scid) [J]. Mycopathologia.

[22] Zhao T, Xu X, Wu Y (2022). Comparison of amphotericin b deoxycholate in combination with either flucytosine or fluconazole, and voriconazole plus flucytosine for the treatment of hiv-associated cryptococcal meningitis: a prospective multicenter study in China [J]. BMC Infect Dis.

[23] Fujioka K, Nagai T, Kinoshita Y (2019). Successful treatment with voriconazole combined with amphotericin b-liposome for fluconazole-resistant pulmonary cryptococcosis after renal transplantation [J]. CEN Case Rep.

[24] Williamson PR, Jarvis JN, Panackal AA (2017). Cryptococcal meningitis: epidemiology, immunology, diagnosis and therapy [J]. Nat Rev Neurol.

[25] Who guidelines approved by the guidelines review committee [M]. Guidelines for diagnosing, preventing and managing cryptococcal disease among adults, adolescents and children living with hiv. Geneva; © World Health Organization; 2022. 35797432

[26] Smith KD, Achan B, Hullsiek KH (2015). Increased antifungal drug resistance in clinical isolates of cryptococcus neoformans in Uganda [J]. Antimicrob Agents Chemother.

[27] Naicker SD, Mpembe RS, Maphanga TG (2020). Decreasing fluconazole susceptibility of clinical South African cryptococcus neoformans isolates over a decade [J]. PLoS Negl Trop Dis.

[28] Setianingrum F, Rautemaa-Richardson R, Denning DW (2019). Pulmonary cryptococcosis: a review of pathobiology and clinical aspects [J]. Med Mycol.

